# Biochemical parameters as early indicators of seagrass transplant’s health

**DOI:** 10.1093/conphys/coag071

**Published:** 2026-09-14

**Authors:** Alize Deguette, Filipe Parreira, Rui Santos, Isabel Barrote, João Silva

**Affiliations:** Centro de Ciências do Mar do Algarve (CCMAR/CIMAR LA), Campus de Gambelas, Universidade do Algarve, Faro 8005-139, Portugal; Centro de Ciências do Mar do Algarve (CCMAR/CIMAR LA), Campus de Gambelas, Universidade do Algarve, Faro 8005-139, Portugal; Centro de Ciências do Mar do Algarve (CCMAR/CIMAR LA), Campus de Gambelas, Universidade do Algarve, Faro 8005-139, Portugal; Centro de Ciências do Mar do Algarve (CCMAR/CIMAR LA), Campus de Gambelas, Universidade do Algarve, Faro 8005-139, Portugal; Faculdade de Ciências e Tecnologia, Campus de Gambelas, Universidade do Algarve, Faro 8005-139, Portugal; Centro de Ciências do Mar do Algarve (CCMAR/CIMAR LA), Campus de Gambelas, Universidade do Algarve, Faro 8005-139, Portugal

**Keywords:** Biochemical indicators, *Cymodocea nodosa*, non-structural carbohydrates, oxidative stress, pigments, restoration, seagrass, transplantation, *Zostera marina*

## Abstract

Seagrass restoration efforts have multiplied in the last years, with very diverse success rates, justifying the need for a better understanding of the physiological aspect of transplant operations. For the first time, biochemical parameters were used as early indicators of transplant’s health. In this study, two seagrass species were transplanted using two distinct modalities per species. *Zostera marina* was transplanted in bundles of five and ten shoots and *Cymodocea nodosa* was transplanted with original sediment in trays and as bare rhizome segments of at least three shoots. Oxidative stress, antioxidant activity, photosynthetic pigment and non-structural carbohydrate (NSC) content in leaves and rhizomes were investigated before transplant and four weeks after transplant. Results revealed some significant method-specific early biochemical responses to transplant for *C. nodosa. Cymodocea nodosa* shoots transplanted in segments showed radical shifts in NSC storage and chlorophyll content whereas antioxidant activity was further triggered in *C. nodosa* transplanted in trays. Shoot density may be an important factor to consider when selecting the best transplant method for *Z. marina*, but no differences were observed in the early biochemical parameters measured. The results of this short-term biochemical monitoring suggest that transplanting *C. nodosa* in trays could be less disruptive for the plant’s biochemistry than bare-rhizome methods. This study provides insights into biochemical responses post-transplantation and emphasizes the need for adaptable restoration strategies. Coupled with transplant survival, morphological parameters and long-term monitoring, biochemical metrics can be used as a sensitive tool to monitor short-term responses in seagrass meadows.

## Introduction

Seagrasses are marine plants that occur in shallow coastal waters in all seas except Antarctica (Green and Short, 2003; Alongi, 2018). When growing in high densities, they form large underwater gardens called seagrass meadows. Seagrass meadows have gained scientific and socio-economic interest in the last decades due to the wide recognition of their high ecological importance and the ecosystem services they provide (Duarte *et al.*, 2013; Mtwana Nordlund *et al.*, 2016). Characterized by their high productivity, seagrasses provide food, nursery grounds and habitat to many species (Boudouresque *et al.*, 2006; Blandon and Zu Ermgassen, 2014), slow down coastal currents, trap sediment and sequestrate and export blue carbon to adjacent ecosystems (Duarte *et al.*, 2005; Fourqurean *et al.*, 2012; Duarte and Krause-Jensen, 2017). Seagrass meadows are threatened worldwide by anthropogenic and climatic pressures, and they have been declining over the last decades (Waycott *et al.*, 2009; Short *et al.*, 2011; Dunic *et al.*, 2021). In Europe, 29% of seagrass meadows disappeared between 1869 and 2016, with the highest decline reported for the subtidal species *Zostera marina* and *Cymodocea nodosa* (de los Santos *et al.*, 2019). Seagrass decline jeopardizes the survival of many species that depend on them and compromises the host of ecosystem services these plants provide. Restoring seagrass meadows is an essential step in the pathway to reverse their decline while also preserving their ecosystem services. Under the UN Decade on Ecosystem Restoration, the United Nations Environment Programme defined restoration as ‘the process of halting and reversing degradation, resulting in improved ecosystem services and recovered biodiversity. Ecosystem restoration encompasses a wide continuum of practices, depending on local conditions and societal choice’. In response to the alarming seagrass loss, seagrass restoration efforts have multiplied, with varying degrees of success (Calumpong and Fonseca, 2001; Paling *et al.*, 2009; van Katwijk *et al.*, 2016; Paulo *et al.*, 2019; Tan *et al.*, 2020; Curiel *et al.*, 2021). Between 2015 and 2022, seagrass restoration represented 21% of seascape restoration projects worldwide, after corals (29%) and mangroves (28%) (UNEP-WCMC, 2022).

Seagrass restoration projects have so far been mostly based on transplanting apparently healthy plants from a donor site to the site to be restored, while some have recurred to seeds, seedlings or plantlets from *in vitro* propagation (Zarranz *et al.*, 2010; van Katwijk *et al.*, 2016; Paulo *et al.*, 2019; Gamble *et al.*, 2021). Contrary to dispersed designs, a clumped configuration can promote sedimentation, alleviate physical stress and allow colonization by other species (Castellanos *et al.*, 1994; Renzi *et al.*, 2019). Neighbour facilitation has previously been shown to enhance the success of seagrass and wetland restoration (Silliman *et al.*, 2015; Valdez *et al.*, 2020). In prior transplant operations, *Z. marina* showed positive density dependence (increased survival and patch expansion), with a high density of shoots promoting growth and reproduction, lowering mortality and increasing restoration success (Olesen and Sand-Jensen, 1994; Almela *et al.*, 2008; van Katwijk *et al.*, 2016; Paulo *et al.*, 2019). Apart from the restrictions posed by the different biotic and abiotic characteristics of donor and receptor sites such as light, temperature, hydrodynamics, grazing, epiphytes and microbial pathogens, which are known to trigger the antioxidant defence system (Michalak, 2006; Szymańska *et al.*, 2017; Rodríguez-Calzada *et al.*, 2019; Chowdhary *et al.*, 2021) and pigment composition (Costa *et al.*, 2015; Roca *et al.*, 2016; Eriander, 2017), transplantation is, itself, stressful for the plants. It disrupts their root and rhizome systems (Wang *et al.*, 2021) and negatively interferes with nutrient absorption and gas diffusion in the aerenchyma throughout the plant (Bloom and Caldwell, 1988). In an intact plant, the oxygen produced during photosynthesis diffuses from the leaves to roots and rhizomes as well as radially, leaking to the surrounding rhizosphere (Pedersen *et al.*, 1998; Colmer, 2003). This oxygen flow to the sediment affects its chemistry and the availability of nutrients, the concentrations of potentially toxic substances, as well as microbial activity and the very existence of microbiome populations that generally favour seagrass survival (Colmer, 2003; Milbrandt *et al.*, 2008; Brodersen *et al.*, 2015). The wounds made in roots and rhizomes during collection for transplant eliminate the barriers these plant structures commonly have (Barnabas, 1996; Scholz *et al.*, 2021), and allow the entrance of microorganisms and toxic substances (Loria *et al.*, 2003; Krupa and Dommergues, 2012). Thus, transplanting is a potential source of physiological stress for seagrasses, not only because of the change in biotic and abiotic conditions but also because wounds inflicted on rhizomes and roots are likely to induce significant changes in the plants’ tissues and consequently in the rhizosphere. Alongside antioxidant activity and pigment composition, non-structural carbohydrate (NSC) content provides a quantitative measure of seagrass response to environmental stress and recovery (Touchette, 2007; Leoni *et al.*, 2008; Jiang *et al.*, 2013). Triose phosphates are the primary product of photosynthesis (McClain and Sharkey, 2019). They can be stored in the chloroplast as starch or exported to the cytosol, where they can be used for the synthesis of other more complex carbohydrates, such as sucrose, glucose and fructose, or used as building blocks for other molecules such as amino acids (McClain and Sharkey, 2019). Sucrose is the main carbohydrate transported along the plant (Kühn *et al.*, 1999; Lemoine, 2000), from sources (e.g. leaves) to sinks (e.g. rhizomes), where it can be stored as starch or readily used for the general plants’ metabolism (e.g. respiration, osmolytes, sensing and signalling) to sustain growth, reproduction, defence and survival (Ruan, 2014; Yoon *et al.*, 2021). While NSCs such as glucose are immediately available substrates for respiration and metabolism (Atkin *et al.*, 2000), other NSCs such as starch constitute reserves that may improve the plant’s chances of survival through winter or when unpredictable environmental stressors occur (Govers *et al.*, 2015).

Successful seagrass restoration depends, among several other factors, on the specific methods selected for transplantation. These cannot be generalized and reproduced everywhere as they strongly depend on the species, location and physical characteristics of the restoration site. This makes each transplantation effort singular, and several methods must be tested to assess the most suitable option for a given species in each location (Campbell, 2002). Transplant success is commonly evaluated through plant survival, biomass, productivity, density, surface coverage and leaf growth (Peralta *et al.*, 2003; Li *et al.*, 2010; Alagna *et al.*, 2019; Oreska *et al.*, 2021; Mokumo *et al.*, 2023; Mourato *et al.*, 2023), but only a few studies have considered and evaluated the effect of transplanting on the plant’s biochemistry (Evans *et al.*, 2018). For the first time, we investigate and compare the early effects of different transplant methods on oxidative stress, antioxidant activity, energy storage and pigment content of *Z. marina* and *C. nodosa*, offering new insights into the plant’s biochemical responses post-transplantation and providing potential early-warning indicators of the transplanted shoots’ health condition.

## Materials and Methods

### Transplant site

Ria Formosa is an 84 km^2^ coastal lagoon located in the Algarve, south of Portugal (36°58′N, 8°02′W to 37°03′N, 7°32′W). It has been a Natural Park since 1987 and a Ramsar and Natura 2000 protected area. It is over 55 km long and a maximum of 6 km wide, making it one of the most important wetlands in Europe (Newton and Mudge, 2003). The lagoon is 2 m deep on average, and its tidal amplitude varies between 3.50 m on spring tides and 1.30 m on neap tides (Instituto Hidrográfico, 1986). It is separated from the Atlantic Ocean by five dynamic barrier islands and two peninsulas; seven channels allow water exchange between Ria Formosa and the ocean. Ria Formosa is an important nursery hotspot and feeding ground for many fish and mollusc species (Andrade, 1985; Ribeiro *et al.*, 2006; Parreira *et al.*, 2021; Erzini *et al*., 2022), which gives the lagoon high ecological and economic importance.

**Figure 1:**
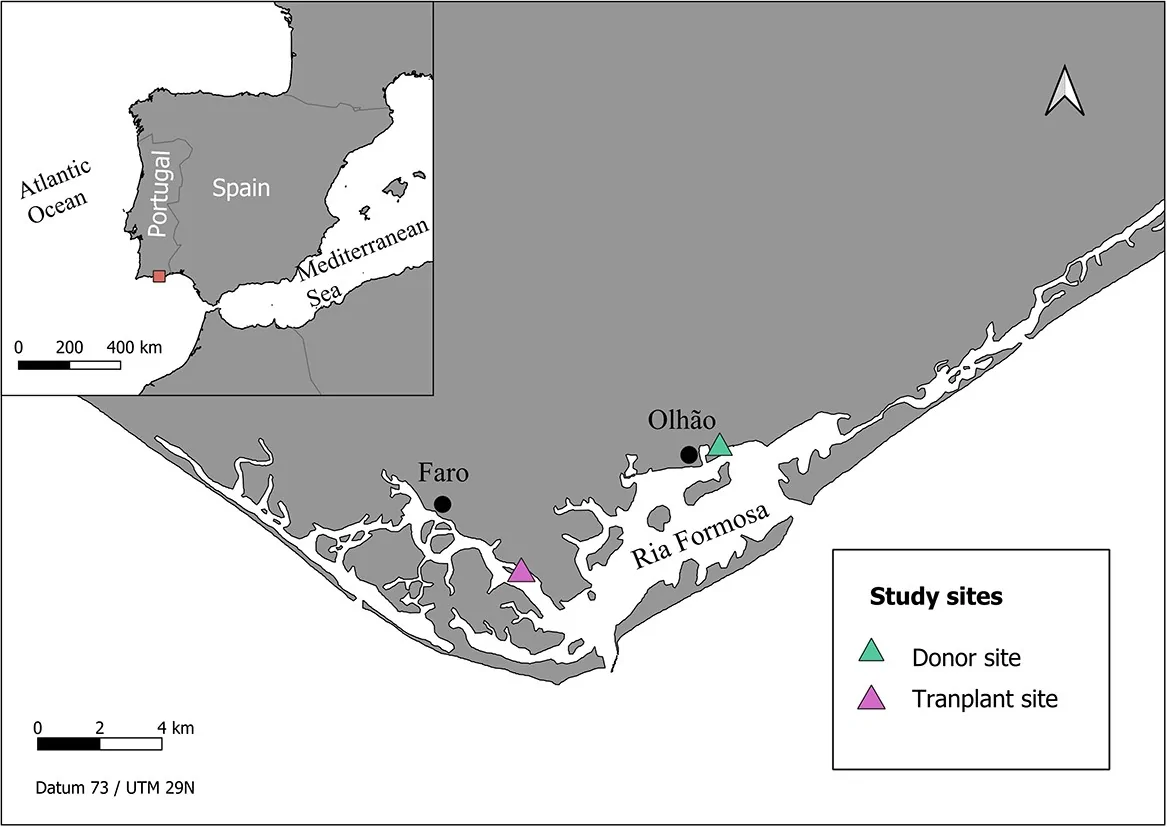
Map showing the location of the donor and transplant sites. Created using QGIS 3.34.9 Development Team, 2020.

The transplant site for this experiment (36°59′33.4″N, 7°54′03.8″W; Fig. 1) is located between the cities of Faro and Olhão, in a secondary channel of the lagoon which has become a seahorse sanctuary in 2020 (Edital 15/2020, Capitania do Porto de Faro). Navigation in this channel is forbidden to preserve seahorses and seagrasses. The transplant area is subject to the natural tidal variations of the lagoon. The margins of the transplant channel are colonized by *Z. noltii* and in the subtidal parts of the channel, patches of *C. nodosa* and *Z. marina* can be found, covering roughly 10–20% of the area. Visually, the density at the transplant site appears to be identical to other parts of Ria Formosa. In the subtidal zone of this channel, a benthic grid with four plots of 3 m^2^ each was built with lines and polyvinyl chloride (PVC) poles to separate different species and transplantation methods. The grid was installed on a sandy-muddy substrate partially covered by *Caulerpa prolifera*, an invasively expanding seaweed. Maximum depth at high tide at this location is 3 m.

### Donor site

**Figure 2:**
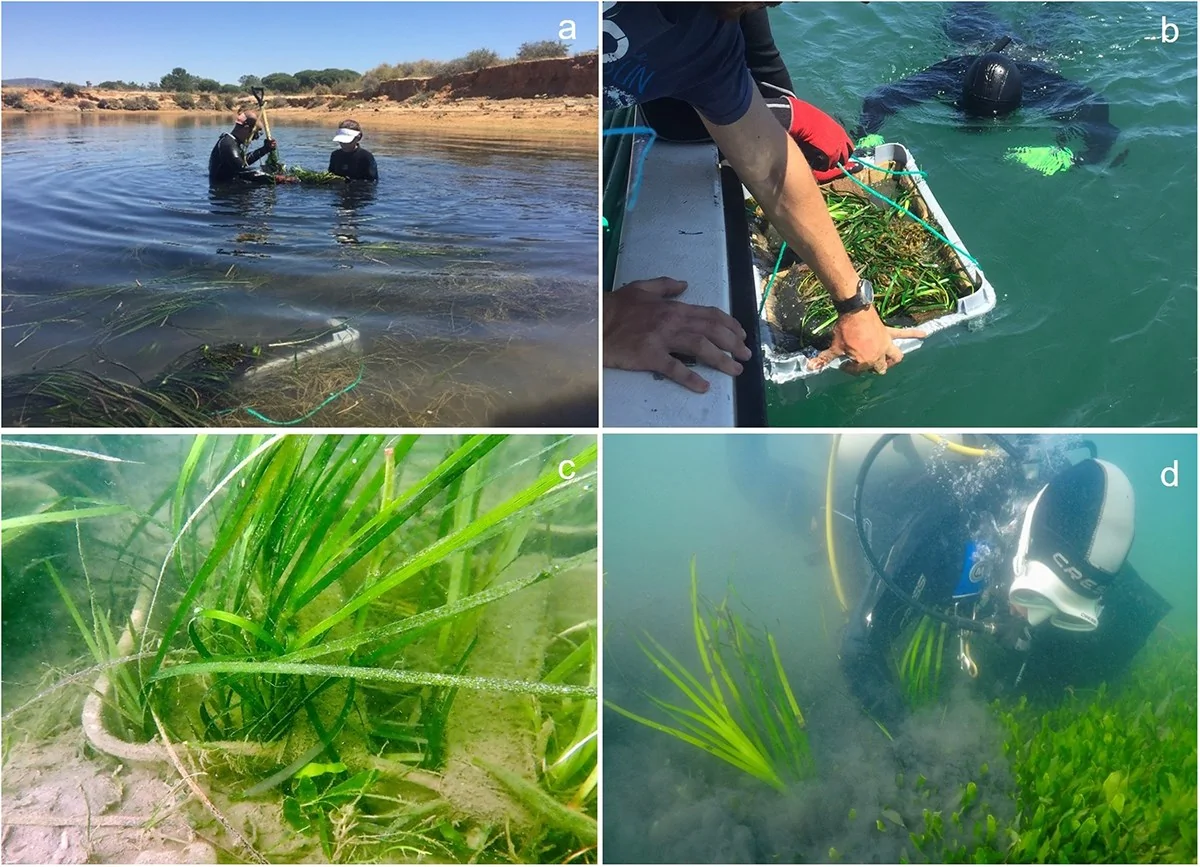
Photographs of seagrass collection and transplantation. (**a**) Collection of *C. nodosa* in trays at the donor site; (**b**) transfer of *C. nodosa* trays from the boat to the transplant site; (**c**) underwater picture of a tray inserted in the transplant plot; (**d**) underwater picture of a diver transplanting bundles of *Z. marina* to the transplant site covered with *Caulerpa*. Photo credits: Filipe Parreira and Alizé Deguette.

To avoid damaging natural meadows, *C. nodosa* and *Z. marina plants* were collected from a semi-natural donor site, in the Pilot Aquaculture Station of Olhão, operated by the Portuguese Fisheries Institute (EPPO/IPMA), located at ~6 km from the transplant site. Seagrasses were collected in a pond (37°01′48.3″N, 7°49′14.0″W) ~1 m deep with sandy-muddy substrate (Fig. 2a). The pond contains natural seawater from Ria Formosa which is renewed with each tide cycle. The water level inside the pond is determined by a dam, where seagrasses are always kept submerged. The area is sheltered, with no significant current or turbulence, no human or boat activity. In this pond, seagrasses grow abundantly, making it an ideal donor site for transplant operations. Sediment type and seagrass morphometric characteristics at the donor and transplant site are visually identical. Preliminary data indicate that temperature, salinity and light conditions are broadly comparable between the collection and transplant site, suggesting that major differences in these variables are unlikely to have driven the results of this study.

### Plant collection and transplantation methodology

**Figure 3:**
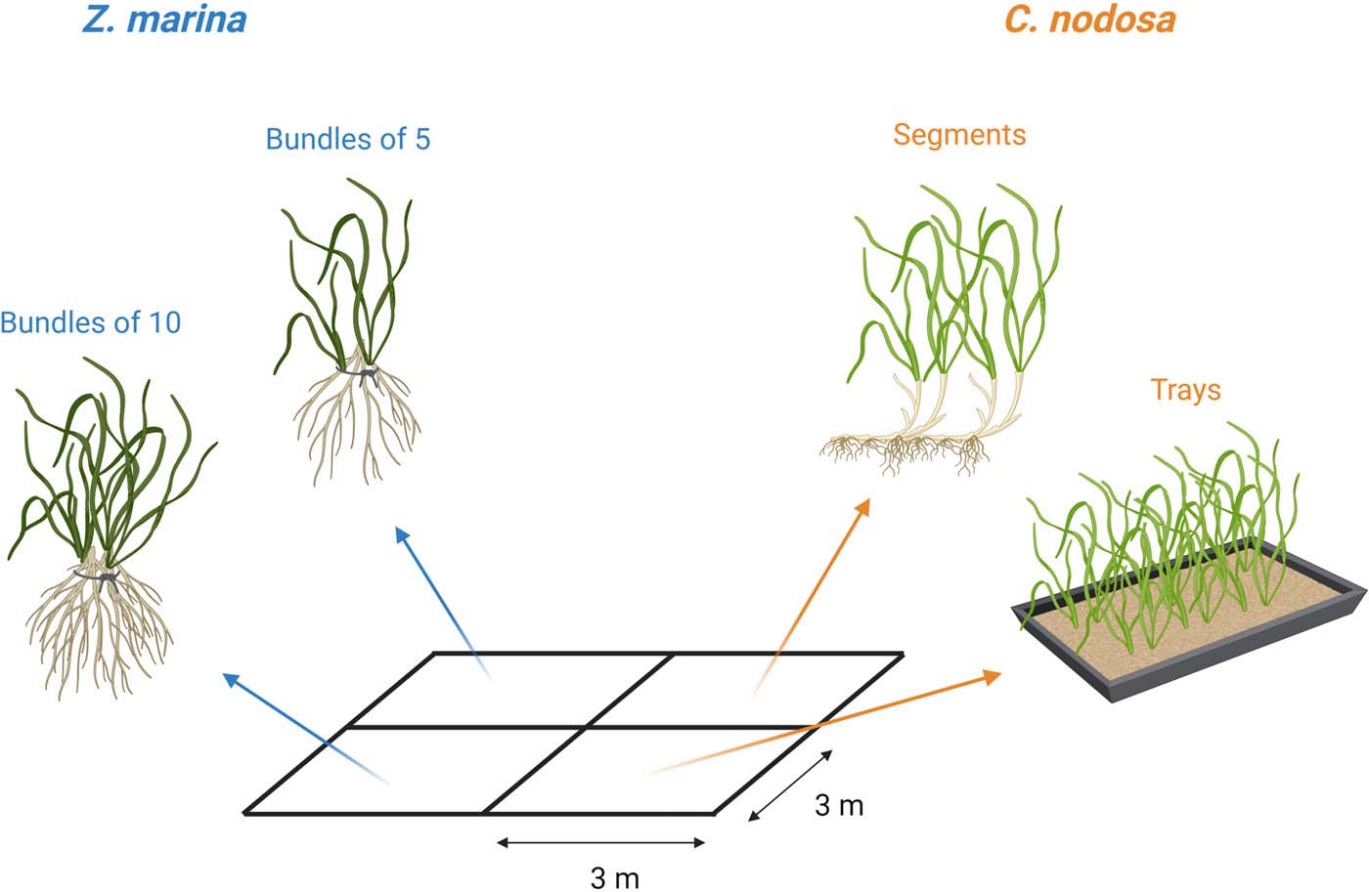
Schematic representation of the transplant plot and representation of the four transplant methods. The plot contains four quadrats of 3 m^2^ across which seagrasses were homogeneously transplanted with the respective modality (*Z. marina*: bundles of 5 and bundles of 10 shoots, *C. nodosa*: segments of at least 3 shoots and biodegradable cardboard trays). Seagrass images were created with BioRender (BioRender.com/d09z118).

The experiment was conducted in June/July 2022. In Ria Formosa, *C. nodosa* tends to develop longer rhizomes than *Z. marina* (>1 *vs* ≤0.3 m, respectively) and with more shoots (often more than 12 *vs* 1, respectively). Because of their distinct morphology, plants were collected and transplanted with different methods (Fig. 3): *Z. marina* plants were collected as bare single-shoot segments (single apical shoot including its own rhizome and roots) and attached in bundles of (i) 5 shoots and (ii) 10 shoots. These bundle sizes were selected based on previous experience with this species ecology and restoration, considering the biology of the species, practical handling during transplantation and the expectation that different initial shoot densities could influence transplant stability and subsequent establishment. *Cymodocea nodosa* was collected as (i) bare apical rhizome fragments of at least three vertical shoots and (ii) as sods with some aggregated sediment placed inside biodegradable cardboard trays. Regardless of the species, the whole plant structure (leaves, rhizomes and roots) was collected with special care to minimize damage. Eight hundred forty *Z. marina* shoots were collected and attached in 84 bundles of 10 shoots (held together with biodegradable coconut fibre thread, ‘10-bundles’). Another 875 shoots were collected to form 175 bundles of 5 shoots (‘5-bundles’). Of *C. nodosa*, 1000 bare segments of at least 3 shoots were collected (‘segments’), and 24 biodegradable cardboard trays (36 × 20 × 11 cm) were filled with a sediment layer ~10 cm deep, extracted with a shovel (Fig. 2a) and containing *C. nodosa* rhizome structures and vertical shoots (‘trays’, 720 shoots). In a quadrat, 750 *C. nodosa* bare segments were transplanted. The remaining collected shoots were transplanted next to the plot. In total, 1715 *Z. marina* and 1720 *C. nodosa* shoots were transplanted in 5 days. Transplantation densities were kept identical to those observed at the donor site. The transplantation effort required five people per day on average, with an average work time of 6 h per day.

Following collection, *C. nodosa* cardboard trays were placed in pairs inside bigger plastic trays (Fig. 2b) and covered with damp towels to keep them shaded and moist to avoid light and drought stress. Trays were carefully transported by boat and carried underwater to the planting plot (Fig. 2b). At the transplant site, divers dug depressions in the sediment, with small shovels, of the same size and depth as the cardboard trays. Divers carefully placed the biodegradable cardboard trays inside the depressions (Fig. 2c) and then levelled the trays and the sediment, as in Paulo *et al.* (2019). Following collection, *Z. marina* bundles were kept submerged in seawater in dark plastic containers and carried to the boat to reach the transplanting site. Divers transplanted *Z. marina* bundles by hand by carefully inserting the rhizomes ~5–10 cm inside the sediment (Fig. 2d). All bundles and segments were transplanted homogeneously across their respective 3 × 3 m quadrat (Fig. 3).

Institute for Nature Conservation regulates the collection and use of seagrasses and other plants for experimental purposes in Ria Formosa Natural Park. Therefore, to comply with national legislation, a permit (Licença n°17/2021/Recolha) was issued for the collection of all the plant material used in this study. All methods were carried out in accordance with relevant national and international guidelines.

### Monitoring and sample collection

Shoots from the donor site were sampled on 23 June 2022, the same day as plant collection (hereafter ‘initial state’). Plants on the transplant site were sampled on 20 July 2022, 4 weeks and 2 days after transplant. Samplings were performed at low tide, around 11 a.m. Approximately 15 plants of each modality were collected to form five replicates of leaf and rhizome material (‘initial state’, ‘bundles of 5’ and ‘bundles of 10’ for *Z. marina*; ‘initial state’, ‘segments’ and ‘trays’ for *C. nodosa*). A total of 60 samples was collected. For each sample, ~100 g of healthy mature leaves and rhizome material were collected, cleaned from epiphytes, rinsed with distilled water and blotted dry before being placed in thin foil bags. Samples were frozen in liquid nitrogen and then stored at −80°C until biochemical analyses.

### Fresh weight/dry weight ratio

For each leaf sample (*n* = 30), ~20 mg of fresh tissue was weighted and dried in an oven at 60°C for 48 h. Fresh weight/dry weight (FW/DW) ratios were calculated and used to express all the descriptors as per gram of dry weight (gDW). Rhizome samples were analysed dry; therefore, no FW/DW ratio was required.

### Antioxidant activity

Antioxidant activity indicators (total phenolic content, TPC; oxygen radical absorbance capacity, ORAC; Trolox® Equivalent Antioxidant Capacity, TEAC) serve as metrics to reflect the plant’s physiological resilience to physiological stress and environmental challenges (Gillespie *et al.*, 2007). TPC measures the total concentration of phenolic compounds—secondary metabolites such as flavonoids and tannins that function as the plant’s primary defence against ultraviolet (UV) radiation, pathogens and environmental stressors (Kumar *et al.*, 2020). ORAC and TEAC measure the actual performance of these molecules. ORAC tracks a plant’s ability to quench peroxyl radicals via hydrogen atom transfer (HAT) (Gillespie *et al.*, 2007), whereas TEAC assesses the ability to scavenge the ABTS^•+^ (2,2′-azinobis-[3-ethylbenzothiazoline-6-sulfonic acid]) radical cation through single electron transfer or mixed mechanisms (Prior *et al.*, 2005).

TPC, TEAC and ORAC were quantified according to Costa *et al.* (2021) and as in Deguette *et al.* (2022). One hundred and fifty milligrams of frozen leaf samples were powdered in liquid nitrogen, suspended in 2.5 ml of hydrochloric acid 0.1 N, kept overnight in the dark under constant shaking at 4°C, centrifuged (4700 ×g, 30 min, 4°C) and brought to a final volume of 5 ml. The supernatant was used to quantify TPC and for ORAC and TEAC assays.

TPC was quantified using the Folin–Ciocalteu method (Folin and Ciocalteu, 1927; Booker and Miller, 1998). Forty-two microlitres of the phenolic extract was added to 0.4 ml Folin–Ciocalteu reagent 0.25 N and 0.4 ml of Na_2_CO_3_ 7.5%. Absorbance was read at 724 nm (Novaspec Plus, Healthcare Bio-Sciences AB, Uppsala, Sweden). Chlorogenic acid was used as a standard, and TPC was expressed as chlorogenic acid equivalents.

TEAC assay quantifies the protection capacity against peroxyl radicals (ROO^•^) through total antioxidant capacity based on a single electron transfer. A cation radical ABTS^•+^ solution was produced by adding 7 mM ABTS to potassium persulphate (2.45 mM final concentration) (Re *et al.*, 1999). Nine hundred and ninety microlitres of diluted ABTS^•+^ solution (absorbance 0.8 ± 0.02 at 734 nm) was added to 10 μl of extract and read at 734 mm. Results were expressed as Trolox® (6-hydroxy-2,5,7,8-tetramethylchromane-2-carboxylic acid) equivalents.

ORAC assay quantifies the protection capacity against peroxyl radicals through HAT. ORAC analysis was done following Huang *et al.* (2002) and Gillespie *et al.* (2007), using ABAP [2,2*^′^*-azobis(2-methylpropionamidine) dihydrochloride] instead of 2,2*^′^*-azobis(2-amidinopropane) dihydrochloride as a lipophilic peroxyl radical generator. One hundred and fifty microlitres of 8.2 × 10–5 mM fluorescein in 75 mM potassium phosphate buffer (pH 7.4) was added to 25 μl of extract, heated to 37°C and read in a Synergy TM 4 multi-detection microplate reader (485 nm excitation filter, 20 nm bandpass, and 528 nm excitation filter, 20 nm bandpass). The reaction was initiated by adding 25 μl of freshly prepared 153 mM ABAP. Results were expressed as Trolox® equivalents.

### Oxidative damage

Malondialdehyde (MDA) is a final secondary product of polyunsaturated fatty acids autooxidation (responsible for cell damage) and enzymatic degradation. Hence, it is considered a valuable indicator of lipid peroxidation under oxidative stress (Hodges *et al.*, 1999). MDA extraction and quantification was performed as in Hodges *et al.* (1999). Three hundred milligrams of frozen leaf tissue were ground in liquid nitrogen and suspended in 5 ml ethanol 80%. After homogenization, the extracts were centrifuged at 3000 ×g for 10 min. One millilitre of supernatant was added to 1 ml of 20% trichloroacetic acid with 0.65% thiobarbituric acid (TBA) and 0.015% butylated hydroxytoluene solution. Two blank solutions were made without TBA or with ethanol 80% instead of sample extract. After mixing well, all samples and blanks were incubated at 90°C for 25 min, cooled down in ice for 15 min, and centrifuged at 3000 ×g for 10 min. The supernatant absorbances were read at 440, 532 and 600 nm, and MDA equivalents were calculated as in Hodges *et al.* (1999).

### Photosynthetic pigments

Photosynthetic pigments were extracted from leaf tissue according to Lichtenthaler and Buschmann (2001). Fifty milligrams of fresh leaves were ground in powder in liquid nitrogen with a small mortar and ~20 mg of sodium ascorbate was added to avoid pigment oxidation. Extraction was done in 5 ml (final volume) of 100% acetone, CaCO_3_-neutralized. The extract was then centrifuged at 4°C, 3000 ×g, 10 min and stored in ice in the dark. Absorbances at 470, 645 and 662 nm were measured in the spectrophotometer. Samples were diluted if the absorbance reading was higher than 0.8. Chlorophylls *a* and *b* and carotenoid concentration in the extracts were calculated as in Lichtenthaler and Buschmann (2001).

### Non-structural carbohydrates

Soluble sugars and starch were extracted from leaf and rhizome tissue and quantified according to Dubois *et al.* (1956) and Burke *et al.* (1996). Sixty milligrams (FW) of leaf tissue were ground in liquid nitrogen in a small mortar. Rhizome samples were freeze-dried (Telstar LyoAlfa 15, Azbil Telstar Technologies S.L.U.) and ground by tungsten ball agitation (Mixer Mill MM400, Retsch) before analysis to ensure samples’ homogeneity. Soluble sugars were extracted from ground leaf (60 mg FW) and rhizome tissue (10 mg DW) in 10 ml of 80% ethanol at 80°C, 30 min and then centrifuged (750 ×g, 5 min). The supernatant was used for soluble sugars quantification and the pellet was kept at −20°C for starch quantification. Soluble sugars were quantified by the phenol sulphuric method using glucose as standard. In a glass test tube, 1 ml of supernatant was added to 1 ml of phenol 5% and 5 ml of sulphuric acid 95% and let to cool down. Absorbance was read at 490 nm and sugar content was quantified as glucose equivalents.

For starch quantification, the pellet was washed three times with distilled water, resuspended in 1 ml of Milli-Q water and incubated at 100°C for 5 min. Starch was hydrolysed by mixing 100 μl of the homogenized pellet suspension with 500 μl of an enzymatic suspension (4 U/ml α-amylase and 2.8 U/ml amyloglucosidase in sodium acetate buffer 0.2 M, pH 4), and incubated overnight at 37°C. The suspension was then centrifuged (750 ×g, 5 min) and diluted 4× in Milli-Q water. Starch was quantified as glucose equivalents as described for soluble sugars.

### Statistical analyses

Each sample was analysed in triplicates and averaged, except for FW/DW ratios (one measurement per sample). Data points that deviated from the upper and lower quartiles more than 3-fold the interquartile range were considered outliers and were excluded from the statistical analysis (Devore and Farnum, 1999). The proportion of excluded observations ranged from 0 to 40% across datasets. Hence, in some datasets, 3 ≤ *n* ≤ 5. Data were tested for normality (Shapiro–Wilk’s test) and homogeneity of variances (Levene’s test). Differences between treatments (initial state, bundles of 5 and 10 for *Z. marina*; initial state, trays and segments for *C. nodosa*) were tested using one-way analysis of variance followed by Tukey HSD *post-hoc* test. *P*-values and test-statistic data are available in Supplementary Materials (Supplementary Tables S1–S6). All tests considered a significance level of *α* = 0.05. All statistical analyses were performed using R Studio software v. 4.3.3. (R Core Team, 2014).

## Results

### Fresh weight/dry weight ratio

The FW/DW ratio of *C. nodosa* leaves was significantly lower in plants transplanted in segments than in trays and in the initial state (Supplementary Table S1). The FW/DW ratio of *Z. marina* leaves did not differ significantly between modalities (Supplementary Table S2).

### Oxidative stress and antioxidant activity

**Figure 4:**
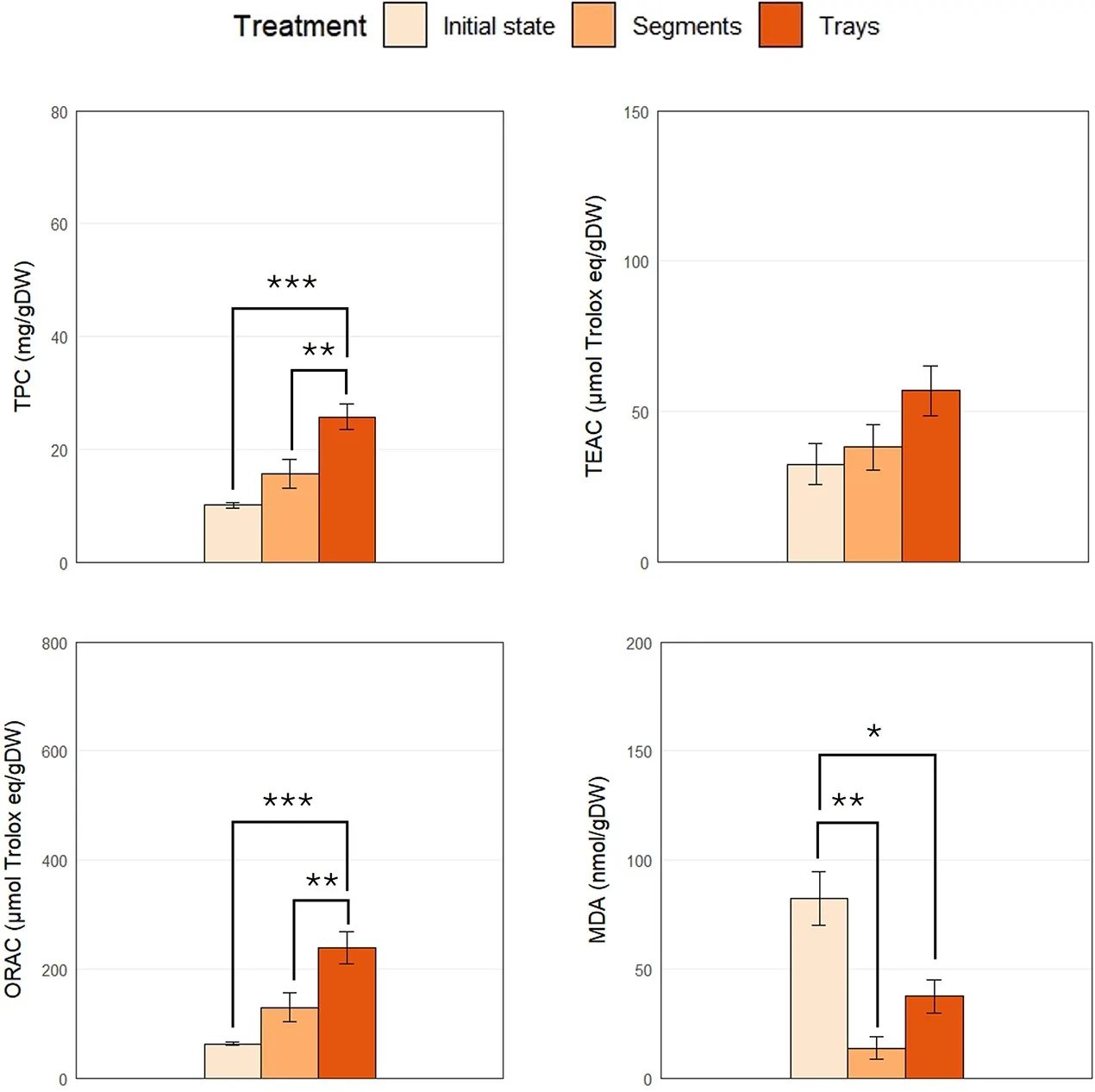
TPC, TEAC, ORAC and MDA content (mean ± SE, *n* ∈ [4,5]) in *C. nodosa* leaves, at the donor site (initial state) and 4 weeks after transplantation with two different transplant methods (segments and trays). ^*^, ^**^ and ^***^ Indicate significant differences with *P* < 0.05, *P* < 0.01 and *P* < 0.001, respectively.

**Figure 5:**
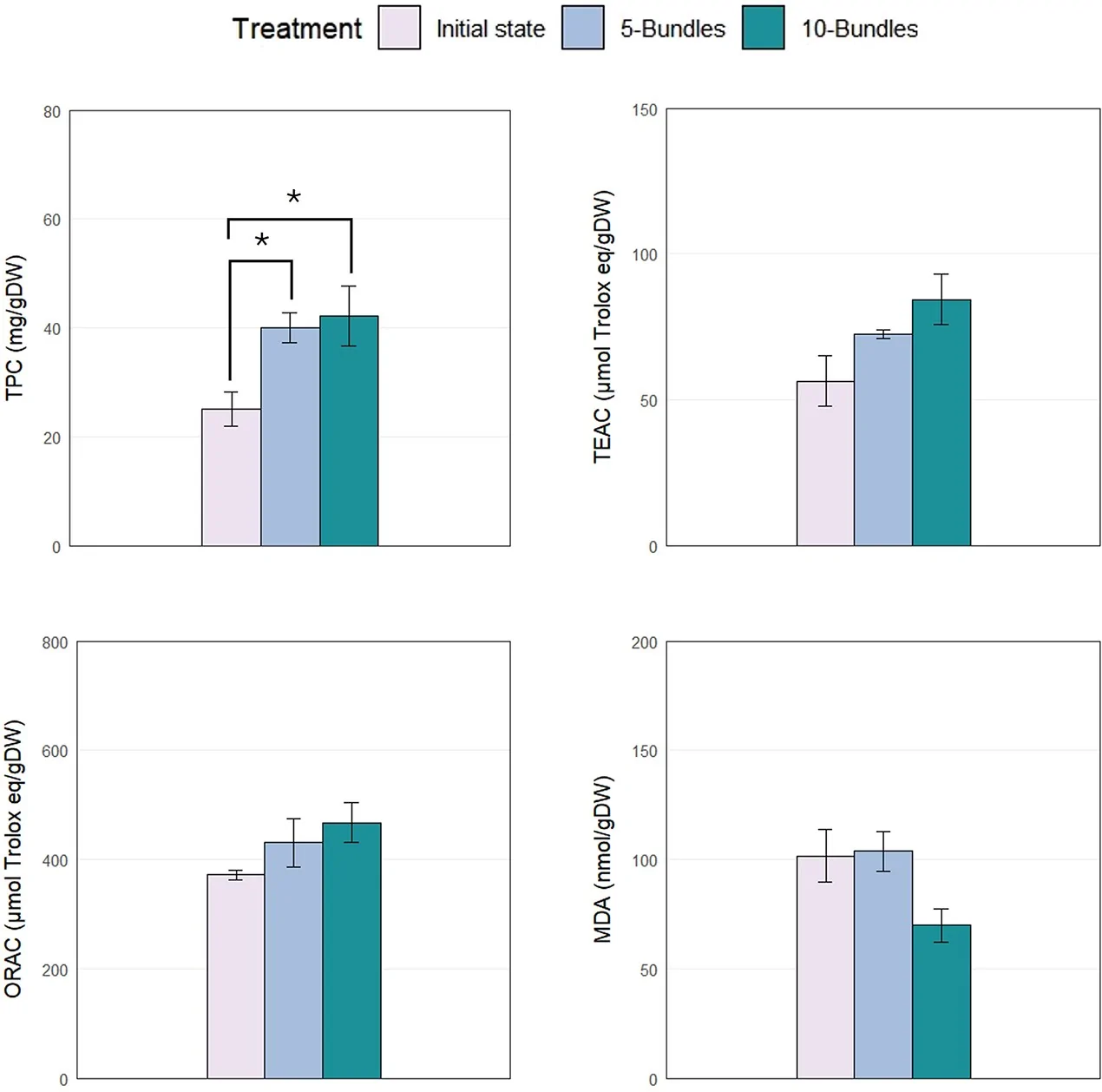
TPC, TEAC, ORAC and MDA content (mean ± SE, *n* ∈ [3,5]) in *Z. marina* leaves, at the donor site (initial state) and 4 weeks after transplantation with two different transplant densities (5 and 10-bundles). * Indicates significant differences with *P* < 0.05.

A general positive trend of antioxidant activity (TPC, TEAC, ORAC) was observed in leaves of both species 4 weeks after transplant relatively to the initial state (Figs 4 and 5). In leaves of *C. nodosa*, TPC and ORAC concentrations increased significantly after transplantation with the tray method compared to the initial state (2.5-fold, *P* < 0.001 and 3.8-fold, *P* < 0.001, respectively; Fig. 4, Supplementary Table S1). TPC and ORAC content was significantly higher in *C. nodosa* transplanted with the tray method than in segments (1.6-fold, *P* = 0.005 and 1.8-fold, *P* = 0.007, respectively; Fig. 4). MDA concentration decreased significantly from the initial state for both segment and tray methods (6-fold, *P* = 0.003 and 2.2-fold, *P* = 0.019, respectively; Fig. 4).

In *Z. marina* leaves, TPC increased significantly 4 weeks after transplant in bundles of 5 and 10 (1.7-fold, *P* = 0.031 and 1.7-fold, *P* = 0.012, respectively; Fig. 5, Supplementary Table S2). No significant difference was observed between transplant densities in oxidative stress nor oxidative damage (MDA).

### Non-structural carbohydrates

**Figure 6:**
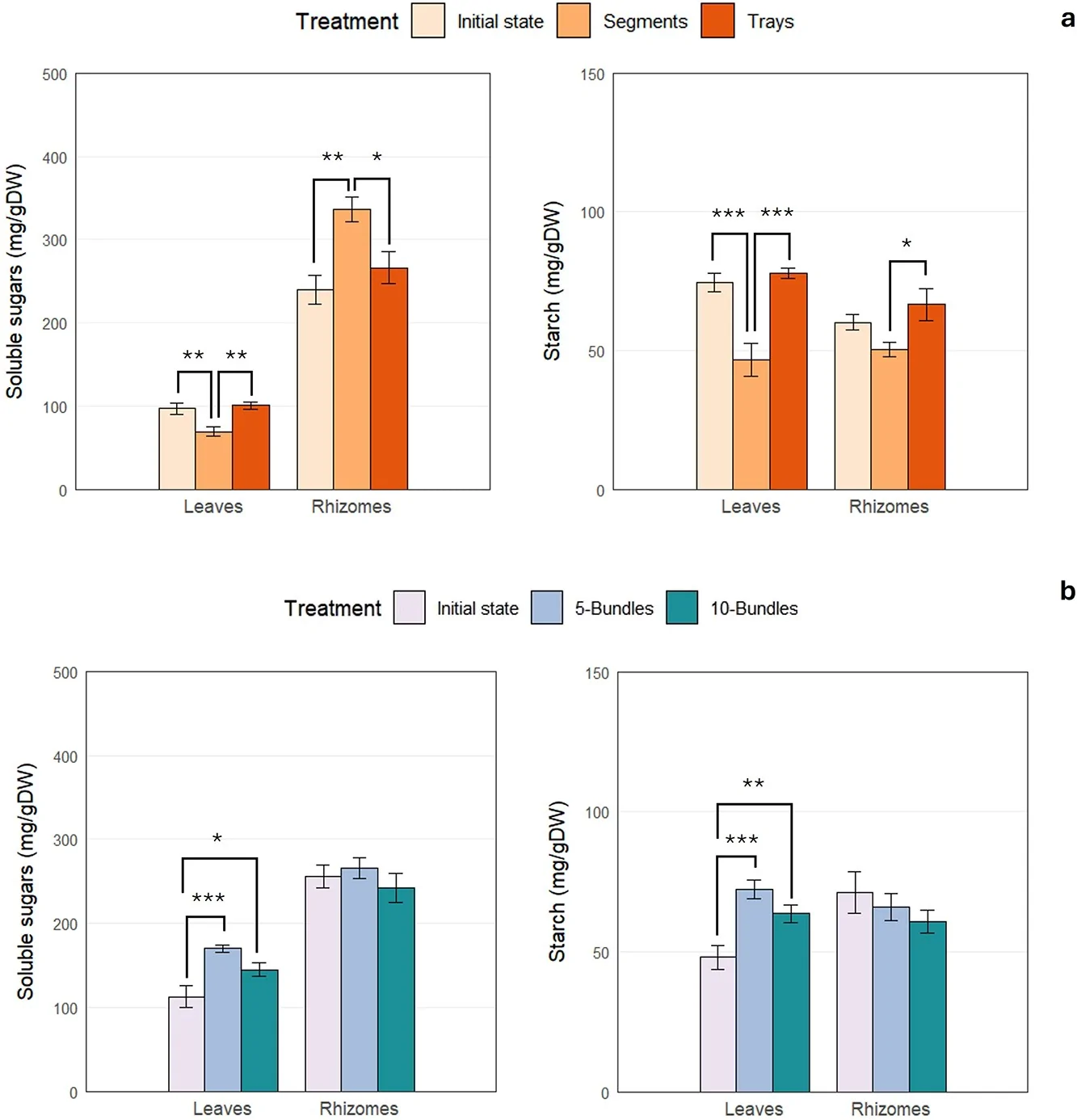
Soluble sugars and starch content (mean ± SE, *n* = 5) in *C. nodosa* (**a**) and *Z. marina* (**b**) leaves and rhizomes at the donor site (initial state) and 4 weeks after transplantation with different transplant methods. Soluble sugars and starch were quantified as milligram equivalents of glucose per gram of dry weight (mg/gDW). ^*^, ^**^ and ^***^ indicate significant differences with *P* < 0.05, *P* < 0.01 and *P* < 0.001, respectively.

The concentration of soluble sugars and starch in the foliar tissue of *C. nodosa* transplanted with the segment method decreased significantly after transplantation (1.4-fold, *P* = 0.005 and *P* < 0.001, respectively; Fig. 6a, Supplementary Table S3). The concentration of soluble sugars in rhizome tissue of *C. nodosa* transplanted with the segment method increased significantly in comparison with the initial state (1.4-fold, *P* = 0.002) and was significantly higher than in plants transplanted with the tray method (1.3-fold, *P* = 0.014; Fig. 6a). *Cymodocea nodosa* transplanted with the tray method contained significantly more starch in leaves and rhizomes, but also more sugar in leaves than plants transplanted in segments (1.7-fold, *P* < 0.001, 1.3-fold, *P* = 0.014 and 1.5-fold, *P* = 0.002, respectively; Fig. 6a).

In *Z. marina* leaves, sugar and starch content increased significantly after transplantation in bundles of 5 (1.5-fold, *P* < 0.001; Fig. 6b, Supplementary Table S4) and in bundles of 10 (1.3-fold, *P* = 0.026 and 1.3-fold, *P* = 0.009, respectively; Fig. 6b). Sugar and starch content in rhizomes did not vary after transplant. There was no significant difference in sugar and starch concentration between transplant densities.

### Photosynthetic pigments

**Figure 7:**
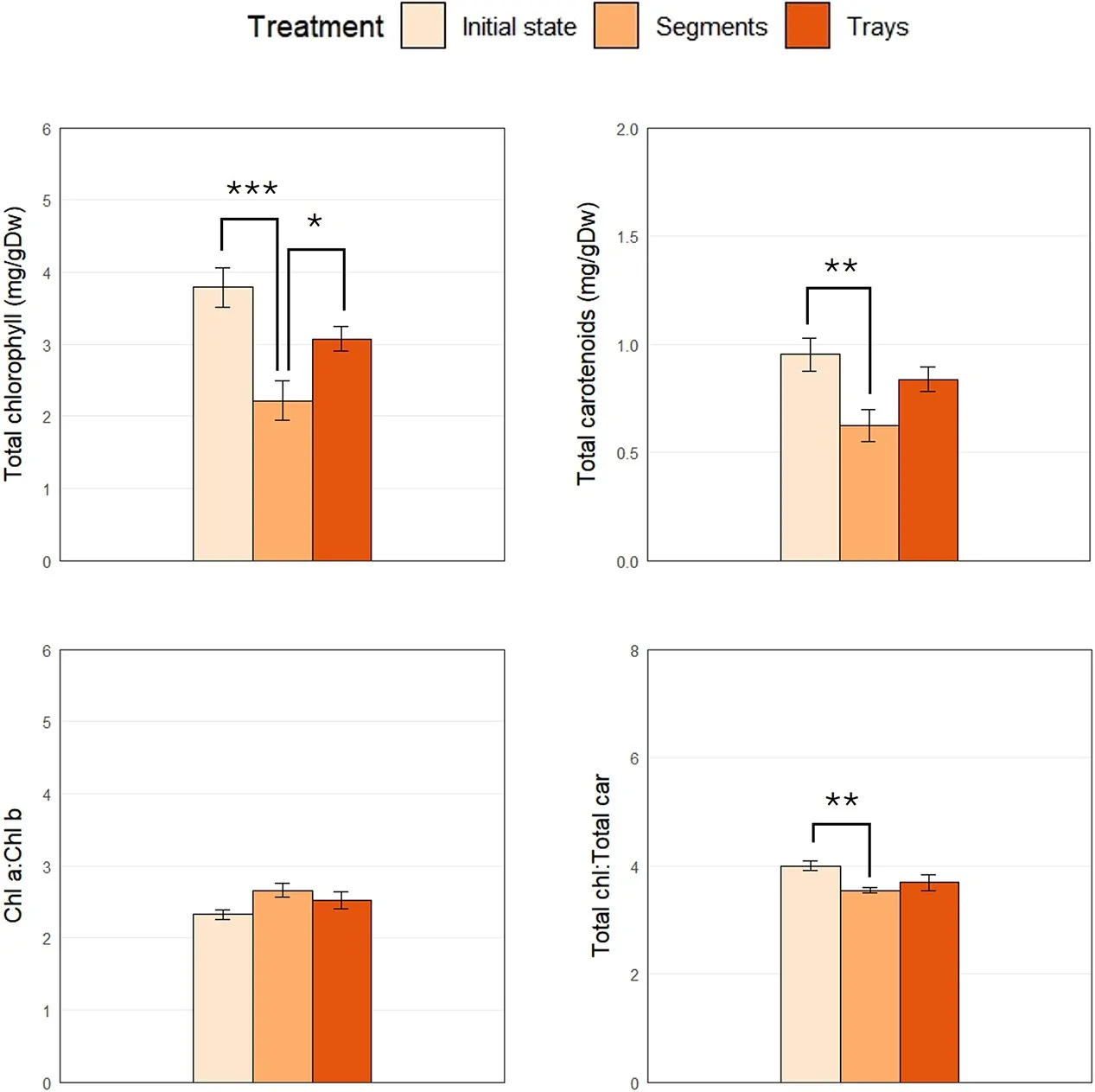
Total chlorophyll and total carotenoids concentrations in milligram per gram of dry weight (mg/gDw), chlorophyll *a:b* ratio (chl *a*:chl *b*), and total chlorophyll to total carotenoids ratio (Total chl:Total car) (mean ± SE, *n* = 5) in *C. nodosa* leaves at the donor site (initial state) and 4 weeks after transplantation with two different transplant methods. ^*^, ^**^ and ^***^ indicate significant differences with *P* < 0.05, *P* < 0.01 and *P* < 0.001, respectively.

The total chlorophyll content was higher in leaves of *C. nodosa* transplanted in trays than in segments (1.4-fold, *P* = 0.028; Fig. 7, Supplementary Table S5). Total chlorophyll, total carotenoids content and total chl:total car ratio decreased significantly in leaves of *C. nodosa* from the initial state after transplant with the segment method (1.7-fold, *P* < 0.001, 1.5-fold, *P* = 0.006 and 1.1-fold, *P* = 0.008, respectively; Fig. 7).

**Figure 8:**
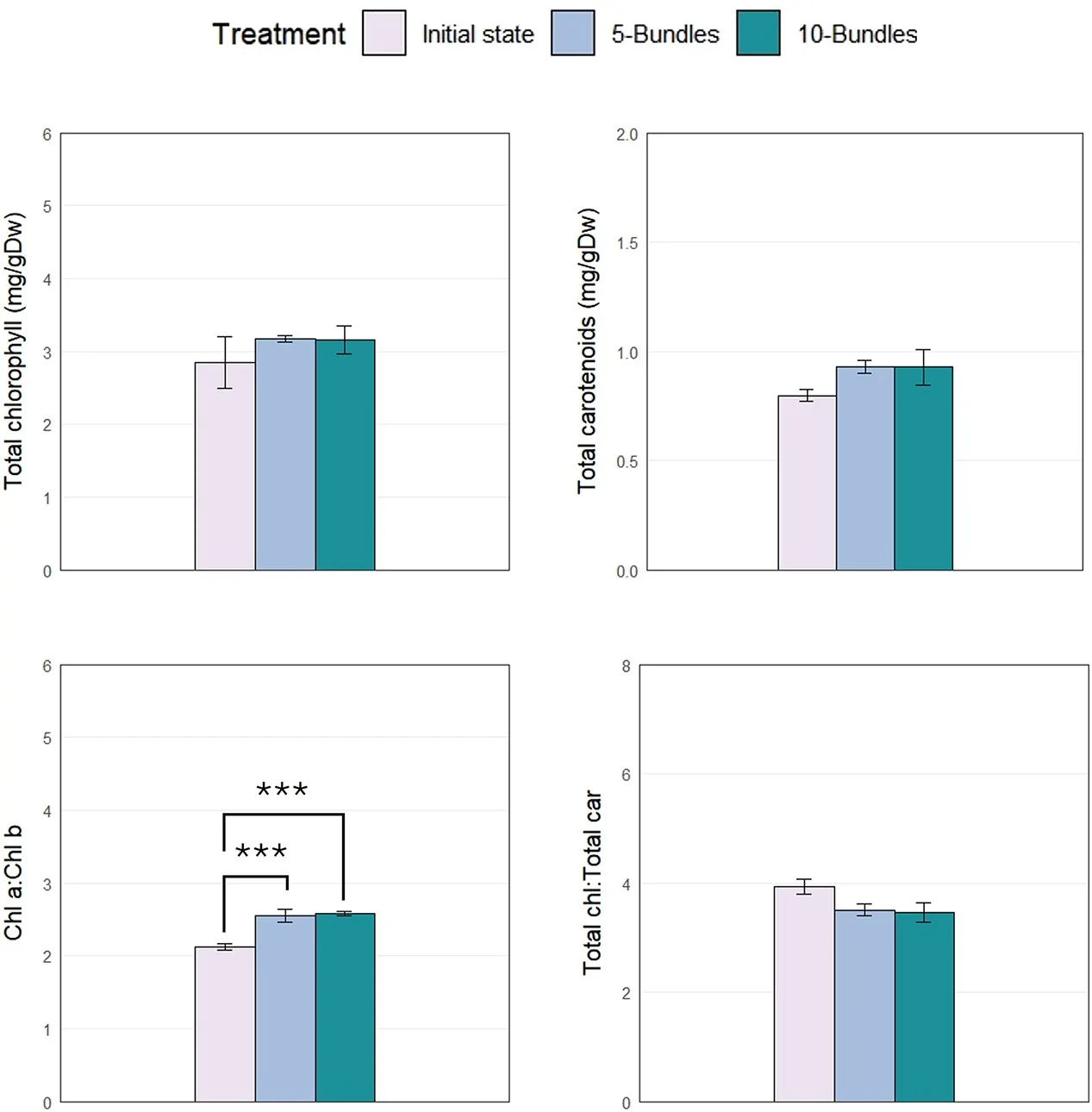
Total chlorophyll and total carotenoids concentrations in milligram per gram of dry weight (mg/gDw), chlorophyll *a:b* ratio (chl *a*:chl *b*), and total chlorophyll to total carotenoids ratio (Total chl:Total car) (mean ± SE, *n* = 5) in *Z. marina* leaves at the donor site (initial state) and 4 weeks after transplantation with two different transplant densities. ^***^ Indicates significant differences with *P* < 0.001.

In *Z. marina* leaves, the chl *a:b* ratio increased significantly after transplant, independently of bundle density (1.2-fold, *P* < 0.001; Fig. 8, Supplementary Table S6). No significant differences in photosynthetic pigment content were detected between bundle densities.

## Discussion

Our results show that the transplant method significantly affected the physiological response of *C. nodosa*. On the other hand, *Z. marina* did not show any significant response to transplant density, according to the biochemical indicators measured.

The distinct early biochemical changes in *C. nodosa* transplanted in trays and segments 4 weeks after transplant suggest that the tray method is more advantageous for the plants than the segment method. Carbohydrates and pigments contents remained at the initial level in tray-transplanted shoots whereas *C. nodosa* transplanted in segments showed significant shifts in carbohydrates storage and lower total chlorophyll content than plants transplanted in trays. On the other hand, tray transplantation triggered increased antioxidant activity compared to segment-transplanted plants. For both species and transplant modalities, MDA did not exceed that of the initial state, suggesting that plants were not subjected to substantial oxidative damage.

Photosynthetic pigments content dropped in the leaves of *C. nodosa* transplanted in segments. While a small dip can be interpreted as a temporary response, significantly lower chlorophyll and carotenoid content may indicate transplantation stress (Genot *et al.*, 1994) or excess photon flux density reaching the leaves (Dennison and Alberte, 1982). The foliar FW/DW ratio of *C. nodosa* transplanted in segments dropped from the initial state and was lower than in *C. nodosa* transplanted in trays. A lower FW/DW ratio indicates a greater proportion of structural/dry matter, or lower tissue hydration compared to the other transplant method. *Cymodocea nodosa* transplanted in segments showed a higher concentration of soluble sugars in rhizomes and a lower sugar concentration in leaves than tray-transplanted ones, indicating that the sugar production rate was lower than the use rate in leaves. Moreover, starch concentration was lower in leaves and rhizomes of *C. nodosa* transplanted in segments than in trays, indicating that sugars were being directly used in metabolism rather than stored as starch. Some soluble sugars are also metabolic signals and act as osmoprotectants to fight against biotic and abiotic stress and provide protection from oxidative damage by scavenging reactive oxygen species (ROS) (Ahmad *et al.*, 2020). Starch stored in the chloroplasts can be hydrolysed, and the hydrolysis products be transported as sucrose to the rhizomes to provide available carbohydrates for growth, respiration or other carbon requirements (Olivé *et al.*, 2007; Serrano *et al.*, 2011; Jiang *et al.*, 2013). We hypothesize that *C. nodosa* transplanted in segments, unlike tray-transplanted shoots, have suffered from severe uprooting and rhizome breakage. NSC reserves were likely used for below-ground antioxidant defence mechanisms and rhizome and root systems re-building. In addition, the intense transplantation stress caused by the uprooting of *C. nodosa* transplanted in segments could have simultaneously resulted in the foliar chlorophyll and carotenoid drop, which in turn suggests the decline in the plant’s photosynthetic activity and consequent decrease of stored starch in leaves and rhizomes to meet the plant’s energy demands. Data on root and rhizome growth and accumulation of antioxidant compounds in belowground parts, together with pulse-amplitude-modulation (PAM) fluorescence or respiration data are needed to support these hypotheses. Root and rhizome breakage caused by shoots handling may also lead to NSC leakage, as discussed in Zimmerman *et al.* (1995), delaying the plants’ establishment post-transplantation. *Cymodocea nodosa* transplanted in trays maintained the same NSC content as the initial state, and rhizomes contained more starch than those transplanted in segments. This supports the idea that the tray method gives plants the advantage of being able to maintain and store energy reserves, notably in rhizomes, which is an asset for survival and growth (Burke *et al.*, 1996; Govers *et al.*, 2015; Soissons *et al.*, 2018).

Antioxidant indicators serve as metrics for evaluating a plant’s capacity to neutralize ROS and mitigate oxidative stress, which can otherwise damage cellular proteins, lipids and DNA (Sharma *et al.*, 2012). Together, the antioxidant activity indicators (TPC, TEAC and ORAC) reflect the plant’s physiological resilience to physiological stress and environmental challenges (Gillespie *et al.*, 2007). As starch and photosynthetic pigments contents were higher in tray-transplanted *C. nodosa*, they have the advantage of reserve energy to deal with extra potential stress and to activate an efficient stress response. In fact, results show that TPC and ORAC were higher in trays than in segment-transplanted shoots. MDA is used as an indicator of cell damage and enzymatic degradation under oxidative stress (Hodges *et al.*, 1999). The absence of increased MDA concentrations, together with the higher antioxidant activity observed in trays likely reflects an enhanced antioxidant buffering capacity rather than increased oxidative stress. This interpretation is further supported by the higher carbohydrate reserves measured in these plants, as the synthesis of antioxidant compounds requires metabolic resources. This gives another advantage to tray-transplanted *C. nodosa* over those transplanted in segments regarding stress tolerance. This is coherent with previous studies which showed that sod transplanting is a successful method compared to seedlings and seed handling or other sediment-free methods (van Katwijk *et al.*, 2016; Paulo *et al.*, 2019).

The metrics studied showed no significant biochemical difference between *Z. marina* transplanted in bundles of 5 and 10 shoots. Both transplant densities appeared to be viable for *Z. marina*, as no significant stress-indicating biochemical shifts were observed. The increased NSC content in leaves and the maintenance of NSC reserves in rhizomes after transplantation could be interpreted as early positive biochemical indications of *Z. marina*’s tolerance to transplantation.

The increase of soluble sugars content in the leaves of *Z. marina* after transplantation could possibly be attributed to an increase of photosynthetic activity to produce new leaves and below-ground biomass, allowing to store energy as starch. Starch storage might be a sign of good health if growth is not negatively affected by transplantation. Data on growth and PAM fluorescence or respiration rate are needed to support this hypothesis. Starch is synthesized when the carbon balance (carbon gain—carbon demand) is positive (Zimmerman and Alberte, 1996; Olivé *et al.*, 2007). Accordingly, *Z. marina*’s photosynthetic activity was probably sufficient to satisfy the plant’s demand for soluble sugars to sustain growth, respiration and storage, this way maintaining NSC reserves in below- ground biomass at initial levels, which offers hope in restoration success (Govers *et al.*, 2015). *Zostera marina* transplants can quickly recover their NSC reserves in the rhizomes following transplant stress, as demonstrated by Gustafsson and Boström (2013) and Eriander (2017). It was shown that transplanting *Z. marina* in too high densities triggers self-shading (Ralph *et al.*, 2007), therefore lowering photosynthetic activity, the production of soluble sugars and/or causing higher stress levels due to competition, which requires a higher sugar consumption. However, *Z. marina* showed no density effect for the metrics studied. The increase in chlorophyll *a:b* ratio from the initial state was consistent with previous studies (Dennison and Alberte, 1982; Bintz and Nixon, 2001) and corresponded to that of high-light conditions. The early biochemical indicators studied here suggest that *Z. marina* transplanted in bundles of 5 and 10 shoots are equally resilient to this transplantation method.

The experimental design of this study limits the ability to attribute the observed changes strictly to the transplantation methods. Plants at the donor site were only sampled at the beginning of the experiment (initial state); however, physiological and biochemical traits in seagrasses can change substantially over short periods of time, particularly during summer. Moreover, the transplanting plot (a single quadrat per transplant modality) could be improved by adding sub-quadrats to avoid quadrat-derived variability. Taking this into account, this study may still demonstrate that biochemical metrics are useful to detect short-term responses post-transplantation, but the conclusions regarding the relative effects of the transplantation methodologies should be taken with caution. Additionally, although the materials used in transplantation methods (cardboard trays, coconut fibre threads) were selected to be as natural as possible, their presence and degradation might still bring physical, chemical or biological alterations to the environment. For example, the cardboard tray can limit the development of roots. Considering the importance of the rhizosphere microbiome of seagrasses for their development and stress-resilience (Tarquinio *et al.*, 2019; Martin *et al.*, 2020; Fuggle *et al.*, 2023; Jongen *et al.*, 2025), it is important to mention that such alterations can potentially be a potential confounding factor to the results observed.

Although it does not indicate transplant success or failure, this study provides novel biochemical insights that expand our understanding of seagrass responses to transplantation stress. Any kind of transplantation induces stress, yet it can be minimized by choosing thoroughly the most adequate methodology. While, until now, restoration success was evaluated using strictly structural and demographic parameters, the inclusion of biochemical indicators has the potential to reveal a faster and more sensitive evaluation of the plant’s health condition in response to transplantation. Biochemical indicators can quickly detect differences in plant’s health condition, yet the inclusion of complementary demographic (e.g. coverage, survival, mortality), morphological (e.g. growth, biomass) and physiological (e.g. PAM fluorescence, respiration) descriptors of seagrass performance is mandatory to provide valuable estimates of plant health and transplantation outcomes. Future research should investigate whether the biochemical parameters measured in this study are directly related to transplantation success. Ultimately, long-term monitoring is needed (e.g. a few months to several years) to evaluate transplant success and assess the perennity of transplanted meadows.

## Supplementary Material

Web_Material_coag071

## Data Availability

The data underlying this article will be shared on reasonable request to the corresponding author.
