# Supplementary material for: Biochemical parameters as early indicators of seagrass transplant’s health
Source: Conserv Physiol. 2026 Sep 14;14(1):coag071. doi: 10.1093/conphys/coag071 (PMC13573650; doi:10.1093/conphys/coag071)
Supplement: Supplementary file 1 [file web_material_coag071.pdf]

Biochemical parameters as early indicators of seagrass transplant's health

Alizé Deguette, Filipe Parreira, Rui Santos, Isabel Barrote and João Silva

Supplementary Materials

Supplementary Table S1. TPC, TEAC, ORAC, MDA contents in *C. nodosa* leaves and fresh weight/dry weight (FW/DW) ratios (mean ± SE), sampled at the donor site (initial state) and 4 weeks after transplant (segments and trays), and results from one-way ANOVAs testing for differences between treatments (F = test-statistic; P = p-value). Different letters indicate, if there are, and for each parameter measured, significant differences among transplant methods. Bold indicates the p-values accounting for significant differences among transplant modalities (<0.05).

	Initial state	Segments	Trays	F	P
**TPC** (mg/gDw)	10.08 ± 0.52 [a] *n=4*	15.65 ± 2.50 [a] *n=5*	25.70 ± 2.58 [b] *n=5*	13.7	**0.001**
**TEAC** (µmol Trolox eq/gDW)	32.37 ± 6.77 *n=5*	38.07 ± 7.64 *n=5*	56.82 ± 8.44 *n=5*	2.8	0.101
**ORAC** (µmol Trolox eq/gDW)	63.05 ± 3.37 [a] *n=4*	129.70 ± 25.85 [a] *n=5*	238.81 ± 29.35 [b] *n=5*	12.8	**0.001**
**MDA** (nmol/gDW)	82.26 ± 13.80 [a] *n=5*	13.79 ± 6.41 [b] *n=3*	37.44 ± 8.74 [b] *n=4*	8.8	**0.007**
**FW/DW**	5.86 ± 0.52 [a] *n=5*	3.61 ± 0.48 [b] *n=5*	5.28 ± 0.18 [a] *n=5*	7.6	**0.007**

Supplementary Table S2. TPC, TEAC, ORAC, MDA contents in *Z. marina* leaves and fresh weight/dry weight (FW/DW) ratios (mean ± SE), sampled at the donor site (initial state) and 4 weeks after transplant (bundles of 5 and bundles of 10 shoots), and results from one-way ANOVAs testing for differences between treatments (F = test-statistic; P = p-value). Different letters indicate, if there are, and for each parameter measured, significant differences among transplant methods. Bold indicates the p-values accounting for significant differences among transplant modalities (<0.05).

	Initial state	5-Bundles	10-Bundles	F	P
**TPC** (mg/gDw)	25.05 ± 3.14 [a] *n=5*	40.04 ± 2.84 [b] *n=4*	42.22 ± 5.53 [b] *n=5*	5.2	**0.026**
**TEAC** (µmol Trolox eq/gDW)	56.40 ± 8.57 *n=5*	72.47 ± 1.50 *n=4*	84.45 ± 8.64 *n=5*	3.6	0.061
**ORAC** (µmol Trolox eq/gDW)	372.14 ± 8.30 *n=4*	430.88 ± 44.27 *n=5*	467.66 ± 36.46 *n=5*	1.7	0.230
**MDA** (nmol/gDW)	101.66 ± 13.50 *n=5*	103.81 ± 9.95 *n=5*	69.90 ± 8.72 *n=5*	3.0	0.086
**FW/DW**	4.48 ± 0.47 *n=5*	5.53 ± 0.14 *n=5*	5.25 ±0.17 *n=5*	3.3	0.07

Supplementary Table S3. Soluble sugars and starch analysis in *C. nodosa* leaves and rhizomes (mean ± SE), sampled at the donor site (initial state) and 4 weeks after transplant (segments and trays), and results from one-way ANOVAs testing for differences between treatments (F = test-statistic; P = p-value). *n=5*. Different letters indicate, if there are, and for each parameter measured, significant differences among transplant methods. Bold indicates the p-values accounting for significant differences among transplant modalities (<0.05).

		Initial state	Segments	Trays	F	P
Leaves	**Soluble sugars** (mg/gDW)	96.72 ± 7.36 [a]	68.89 ± 5.56 [b]	100.59 ± 4.07 [a]	8.8	**0.004**
	**Starch** (mg/gDW)	74.62 ± 3.39 [a]	46.58 ± 5.99 [b]	77.84 ± 1.88 [a]	17.4	**0.000**
Rhizomes	**Soluble sugars** (mg/gDW)	239.3 ± 17.50 [a]	336.14 ± 14.88 [b]	266.05 ± 18.9 [a]	8.5	**0.005**
	**Starch** (mg/gDW)	60.2 ± 2.71 [ab]	50.32 ± 2.50 [a]	66.57 ± 5.89 [b]	4.2	**0.042**

Supplementary Table S4. Soluble sugars and starch analysis in *Z. marina* leaves and rhizomes (mean ± SE), sampled at the donor site (initial state) and 4 weeks after transplant (bundles of 5 and bundles of 10 shoots), and results from one-way ANOVAs testing for differences between treatments (F = test-statistic; P = p-value). *n=5*. Different letters indicate, if there are, and for each parameter measured, significant differences among transplant methods. Bold indicates the p-values accounting for significant differences among transplant modalities (<0.05).

		Initial state	5-Bundles	10-Bundles	F	P
Leaves	**Soluble sugars** (mg/gDW)	112.50 ± 12.68 [a]	169.85 ± 4.49 [b]	144.67 ± 7.83 [b]	10.2	**0.002**
	**Starch** (mg/gDW)	47.99 ± 4.19 [a]	72.33 ± 3.27 [b]	63.56 ± 3.15 [b]	11.9	**0.001**
Rhizomes	**Soluble sugars** (mg/gDW)	255.82 ± 13.56	265.86 ± 12.01	242.23 ± 17.81	0.6	0.538
	**Starch** (mg/gDW)	71.23 ± 7.42	65.97 ± 4.82	60.64 ± 4.15	0.9	0.439

Supplementary Table S5. Photosynthetic pigments content in *C. nodosa* leaves (mean ± SE), sampled at the donor site (initial state) and 4 weeks after transplant (segments and trays), and results from one-way ANOVAs testing for differences between treatments (F = test-statistic; P = p-value). *n=5*. Different letters indicate, if there are, and for each parameter measured, significant differences among transplant methods. Bold indicates the p-values accounting for significant differences among transplant modalities (<0.05).

	Initial state	Segments	Trays	F	P
**Chl *a*** (mg/gDw)	2.65 ± 0.19 [a]	1.61 ± 0.20 [b]	2.20 ± 0.13 [a]	8.7	**0.005**
**Chl *b*** (mg/gDW)	1.14 ± 0.09 [a]	0.61 ± 0.08 [b]	0.87 ± 0.04 [c]	13.9	**0.000**
**Total chlorophyll** (mg/gDW)	3.79 ± 0.27 [a]	2.22 ± 0.27 [b]	3.08 ± 0.16 [a]	10.5	**0.002**
**Total carotenoids** (mg/gDW)	0.95 ± 0.08 [a]	0.62 ± 0.07 [b]	0.84 ± 0.06 [ab]	5.6	**0.019**
**Chl *a:b***	2.32 ± 0.06	2.66 ± 0.10	2.53 ± 0.12	3.1	0.082
**Total chl:Total car**	3.99 ± 0.09 [a]	3.54 ± 0.05 [b]	3.69 ± 0.14 [ab]	5.1	**0.024**

Supplementary Table S6. Photosynthetic pigments content in *Z. marina* leaves (mean ± SE), sampled at the donor site (initial state) and 4 weeks after transplant (bundles of 5 and bundles of 10 shoots), and results from one-way ANOVAs testing for differences between treatments (F = test-statistic; P = p-value). Different letters indicate, if there are, and for each parameter measured, significant differences among transplant methods. Bold indicates the p-values accounting for significant differences among transplant modalities (<0.05).

	**Initial state**	**5-Bundles**	**10-Bundles**	**F**	**P**
**Chl *a*** (mg/gDw)	1.93 ± 0.24 *n=5*	2.27 ± 0.01 *n=4*	2.29 ± 0.13 *n=5*	1.5	0.275
**Chl *b*** (mg/gDW)	0.92 ± 0.12 *n=5*	0.90 ± 0.03 *n=5*	0.87 ± 0.06 *n=5*	0.1	0.903
**Total chlorophyll** (mg/gDW)	2.85 ± 0.35 *n=5*	3.17 ± 0.04 *n=4*	3.16 ± 0.19 *n=5*	0.5	0.605
**Total carotenoids** (mg/gDW)	0.80 ± 0.03 *n=4*	0.93 ± 0.03 *n=5*	0.93 ± 0.08 *n=5*	1.7	0.229
**Chl *a:b***	2.12 ± 0.05 [a] *n=5*	2.56 ± 0.10 [b] *n=4*	2.58 ± 0.03 [b] *n=4*	16.8	**0.000**
**Total chl:Total car**	3.94 ± 0.14 *n=5*	3.51 ± 0.12 *n=4*	3.46 ± 0.18 *n=5*	3.1	0.088
